# Some Notes upon Soft-Shelled Turtles, and the Anatomical Vagaries of Aspidonectes Spinifer

**Published:** 1888-01

**Authors:** G. Archie Stockwell

**Affiliations:** Port Huron, Michigan


### Art. IV.—NOTES UPON SOFT-SHELLED TURTLES AND THE ANATOMICAL VAGARIES OF ASPIDONECTES SPINIFER.

BY G. ARCHIE STOCKWELL, M. D., F. Z. S.
*Port Huron, Michigan.*

Mr. George Brown Goode of the Icthyological Commission, in the "History of Useful Aquatic Animals", enumerates no less than six species of *Trionychidæ* as peculiar to the United States and Canadas, embraced by the two genera

*Aspidonectes* and *Amyda*—the *Trionyx* of Lesson, Cuvier and others.

In the Northwestern States, Michigan and Wisconsin more particularly, and in the Province of Ontario, *Aspidonectes spinifer* and *Amyda mutica* (it is with these two alone that I have to deal) are found in comparative abundance, and of various sizes, from a few inches up to two feet and more, with a weight of from three to eighteen pounds. Not only are they encountered in the same general areas, but, not infrequently, are observed inhabiting the same ponds and streams, and exhibiting almost identical habits. The latter is more generally distributed, however, and is the species commonly indicated when the term "soft shelled turtle" is employed, as *Aspidonectes*, from some incomprehensible cause, is restricted to waters possessing specific topographical features and relations. Nevertheless both are inhabitants of the Great Lake Basin from the St. Lawrence to the upper Mississippi, *A. mutica* extending its range beyond, to the west, into the Yellowstone Valley—if Smithsonian authorities may be credited—while *A. spinifer* has been observed by Canadian naturalists as far north as Lake Athabasca Strange to say also, while Old World *Trionychidæ* are almost exclusively confined to regions south of the Twenty-first Isothermal, the reverse, in the main is true of the American branch of the family.

The generic differences observed between *A. mutica* and *A. spinifer* are somewhat marked. Both present the same flat, almost orbicular, body, enclosed between the usual osseous shields which, with all this family, are found with flexible margins as the result of a leathern skin that replaces the horny epidermis peculiar to other Testudinata; hence the vulgar pseudonym of "soft shelled." The marginal ossicles in *A. mutica* are rudimentary, in *A. spinifer* altogether wanting; and in the latter nearly one-half the length, and more than one-fourth the breath of the upper shell or carapace is composed of fibrous membrane—*not* cartilage, as is sometimes claimed. Neither does the carapace as formed by continuous ossifications of the upper surface of the dorsal and sacral vertebræ and the sub-dermal and intercostal fibrous membranes, wholly embrace the ribs as with marine turtles ; on the contrary, the latter are usually to be traced throughout their length from the articulation to the distal extremity—that is to say : Instead of being blended with the sub-dermal and intercostal ossifications and appearing as mere osseous ridges of the same, they have all the semblance of being the primary osseous structures (as they really are) to which a roof has been subsequently added. The most marked result of this defect of ossification is to be observed in *Aspidonectes spinifer*, where the eighth pair of ribs is wholly separate from those anterior, and held in position by dense fibrous investment alone, a condition entirely at variance with commonly accepted teachings. Cuvier, speaking of the genus *Aspidonectes* under the obsolete title of *Trionyx*, declares they are possessed of "eight pairs of ribs united by *sutures*, which in these, as well as in marine turtles, are *not* dilated to their outer ends" but the dilation "is extended accordingly as the age of the reptile increases."*

As no traces of a sternum are ever observed in *Testudinata*, the plastron in *Trionychidæ* is no exception to the rule, being merely fibrous ossifications arranged in four pairs on either side of a central piece, and without relation in form or arrangement to the ribs or carapace ; and with *A. spinifer* it is not ossified across its middle. Neither is the pelvis united with the carapace or the plastron, though both it and the pectoral arch, almost identical in form, have the appearance of holding a most anomalous position, and of being situate inside the skeleton of the trunk. Since the plastron does not correspond to the sternum of the higher orders but is part of the derma, such anomaly cannot exist on the ventral side ; and as to the dorsal, Professor Huxley assures us in fœtal *Testudinata* the pectoral and pelvic arches are placed, respectively, in front and behind and external to the ribs the same as in other *Vertebrata*. It is only as development advances that the first costal plate extends over the scapulæ and the hinder costal plate over the ilia. The pectoral arch is ossified in such a manner the

***Ossemens Fossiles:* tome ix., p. 398.

scapula and *pre*-coracoid unite as one bone, leaving the coracoid distinct, though its free end is usually united to that of the pre-coracoid by a fibro-cartilaginous band. Of course there is no clavicle except as the epiplastra and entoplastron may represent this bone. The pelvis on the contrary contains the usual number of bones, the pubes (largely developed) and the ischia meeting to form a long symphysis; but it does not unite with the carapace or plastron.*

The two forms under consideration alike exhibit a general dark olive-brown hue that is nearly uniform in *A. mutica*, but in *A. spinifer* is diversified by mottlings upon the carapace that extend to the legs and feet; and the head and neck of the latter are longitudinally striped with two shades of olive-green. *A. spinifer* also has the anterior carapace studded with spines and tubercles, largest and most abundant in the female, who, all other things equal, is nearly or quite double the size of the male; a blunt keel is likewise observed along the median line sloping uniformly to the sides. Both possess stout, compact heads, with long, flexible, tubular, swine-like snouts, but the former has the nostrils more oval, sub-terminally situated, and lacking the protuberant ridges that, arising from either side of the septum are seen occupying the terminal semi-lunar nasal apertures of *Aspidonectes*. The carapace in *Amyda*, moreover, is conspicuous for a marked depression in lieu of a keel or ridge along the median line, and for the absence of spines and excrescences.

I have before remarked the identity of habits as observed in the two species. Both are aquatic, carnivorous, and voracious, the inveterate foes of weaker forms of life, including fish, batrachians, fish-fry and spawn, etc.; they are likewise insatiate devourers of most forms of animal refuse and excreta. Though commonly accredited with partaking of vegetable matter, and even with ravaging potato fields by

*This is quite different from the condition in *Chelydrida* and *Chelonia*, with whom it is common to find union by *synchondrosis* or *anchylosis* between the posterior costal plate and the ilia, and again between the pubes, ischia, and xiphisternal plates.

feeding upon the unripe tubers and tender vine-stalks, such statements are best taken *cum grano* for two reasons: First, the relations of the intestinal tract are such as to indicate a strictly carnivorous diet. Second, the peculiar conformation presented by feet and paws, though conducive to natorial progression, inhibits extended terrestial locomotion, so much so that I doubt if either (*Aspidonectes* more especially), ever wanders a dozen yards from the marshy surroundings of its habitat, save, perhaps, in June or July when it becomes necessary to seek ground suitable for the placing of its eggs to advantage and where they will most surely receive transmitted solar heat. Jordan, in his "Vertebrates of the Northern United States," accredits all *Trionychidæ* with five toes densely webbed, three alone armed with claws; but this is true neither of *A. spinifer* or *A. mutica.* The former exhibits but four toes, three with claws, and one rudimentary and oftentimes wholly wanting; and the latter, though possessed of the full complement, exhibits two in a most undeveloped condition; the phalanges in both, moreover, present little uniformity and no constancy.

My earliest acquaintance with *Aspidonectes* was obtained as a pupil and assistant of the late Abram Sager, while the latter was State Zoologist of Michigan; and the information thus secured, aided by the notes made (and more recently verified and elaborated) are the basis on which this paper is founded. But before discussing the individual peculiarities of the species, let me note an anomaly of distribution that is apparently inexplicable:

Though careful search was repeatedly made, neither in Michigan or Ontario were we able to detect any evidence of it in waters that had their source upon the eastern or northern slopes of the great water sheds, however favorable they appeared to such existence. Inhabiting the streams of the Upper Peninsula of Michigan flowing into the lake of that name, it is absent in those flowing into Lake Superior. Again, while the species is found in every stream of the northern portion of the Lower Peninsula, emptying to the west and south of Fox Point, it is nowhere known to Lake Huron, save upon the opposite Canadian shore. The Manistee and Au Sable Rivers, each with a branch heading in the same district and within a brief half mile, the first a tributary of Lake Michigan and the other of Lake Huron, render this more marked since *Aspidonectes* is found at the very source of the former, and undetected in the latter, though persistent search has been made throughout nearly one hundred miles of its tortuous course. A frequenter of Lake St. Clair, it is had in the Thames, a Canadian tributary, but not in the Clinton, which empties on the opposite side of the Lake Dr. Sager observed much the same in Wisconsin and Illinois, finding the species in the waters to the north and east of Green Bay (but not to the south), and in streams flowing into the Mississippi and Ohio.

The head of *A. spinifer* differs little in osteology from others of the same order and family ; all the bones save the inferior mandible and hyoidean arch are immovably united. The eyes possess two lids, with the customary nictating membrane, closing transversely ; and the tympanic membrane is hidden by integument.

The jaws are trenchant, the lower composed of twelve bony pieces, six to each side, their alveolar edges distinctly serrated and sheathed by a substance presenting the general aspects of bone, but under the microscope revealing the angular cells peculiar to horny tissue ; a fold of skin also simulates lips. The palatines are firmly united to the pterygoids behind, with the vomer in front and above, and with a downward prolongation develop a short, stout, palatine plate that joins with the expanded edge of the vomer to bound the posterior nares. The hyoidean apparatus is made up of a broad cartilaginous plate with four ossified cornua, two anteriorly (the longer), and two posteriorly ; these cornua, however, have no connection with the skull.

Just as the head is small, relatively, as compared with the body, so is the cranial cavity to the head ; decidedly flattened, it is also broadened posteriorly by large temporal fossæ, each partially concealed beneath a bony arch. The brain, again, is disproportionate to the size and relations of the spinal nervous system. The hemispheres, oval in form, more or less elongated according to size and age of the individual, within are hollowed by a single ventricle, and without smooth, are wanting in convolutions or traces thereof. Not only is the spinal marrow excessively developed with nerve filaments proportionately larger and coarser than in higher Vertebrates, but the nuclei of the inferior horns of the cervical enlargement are nearly or quite double those of the posterior enlargement. This is scarcely corroberative of the hypothesis of Dr. J. J. Mason, as promulgated at a recent meeting of the American Neurological Society, that in all *Vertebrata* the diameters of the nuclei of the respective enlargements are the gauge of the powers of the two extremities.

The tongue is short, triangular, but not fleshy, its surface exhibiting great numbers of delicate, bifid, fringe-like processes, semi-regularly arranged in rows, and closely resembling the internal cilliated respiratory apparatus of the tadpole; and these are continued upon the fauces, rima-glottidis, hyoidean arch, and throughout the greater portion of the gullet. This is not at all in consonance with the teachings of comparative anatomists as drawn from the study of other *Testudinata*, since they are wont to describe the œsophagus as presenting "longitudinal folds and mucous crypts"; the lower portion, however, is longitudinally plicated, apparently from contraction of its muscular coat.

The alimentary canal also varies in proportions and relations from general description. T. Rymer Jones declares it to be shorter in soft shelled turtles than in other *Testudinata*, the great and small intestines approximating in length; and on a subsequent occasion he employs the following uncertain language in describing the colon: "It is not longer than the small" (intestine). Cuvier also asserts the relative length of the primæ viæ, as based upon his observations in *Emydidæ*, to be "*five times the length of the body.*" But in the largest specimen of *Aspidonectes* examined, a female with a total length of body of *twenty-*

*five and one-half* inches, the digestive apparatus extended to its utmost measured precisely *four feet*, of which the œsophagus claimed *nine*, the stomach *six*, the small intestine *twenty-seven*, and the colon and cloaca together *six* inches. Here the colon was scarce one-ninth of the primæ viæ, and only about one-fifth the length of the lesser intestine; yet error might arise from the fact the two are almost continuous, without flexion or angle, the junction being marked merely by a slight valvular constriction. The colon, however, is a trifle greater in diameter, less muscular, and consequently more distensible.

The stomach appears less as a specific organ than an extension of the duodenum connecting with the gullet, and is an elongate transverse canal, flexed upon itself, partaking somewhat of the muscular characters of the œsophagus. Even its mucous lining is not arranged in folds, though there is a tendency to plication of the cardiac portion; but it is remarkably and beautifully reticulated, the same being continued throughout the intestines to the cloaca.

The most prominent feature on removing the plastron and exposing the thorax and viscera is the appearance of the heart, if it still be in action (and its contractions and the vermicular movements of the bowels are perfect even when thirty-six hours has elapsed since decapitation), which simulates the four chambered organs of warm blooded higher Vertebrates; the deception is so perfect that it can only be exposed by the knife, and is due to the strong, partially muscular, and partly cartilaginous septum, that divides the common ventricle into a large (left) and small (right) moiety or cavity. The left moiety receives the blood from the auricles, and in consequence of the elongate form of the ventricle as a whole, and the enormously developed auriculo-ventricular valves of the right side that project into it, is virtually subdivided at the moment of auricular systole, forming a third ventricular cavity. The left *sub-division* is filled with arterial blood from the left auricle, and hence is distinguished as the *cavum arteriosum*; the right *sub-division* receiving venous blood from the right auricle is, correspondingly termed *cavum venosum* ; and the original primary right moiety of the common ventricle (as formed by the septum attached between the origin of the left aortic arch and that of the pulmonary artery, its free edge looking toward the dorsal surface of the heart) from which is given off the pulmonary artery, obtains the title of *cavum pulmonale.* No arterial trunk is given off from the left *sub-division,* but two aortic arches are observed at the right-hand end of the right sub-division, one passing to the left side, the other to the right, and crossing one another, owing to the fact the first has its origin more to the right than has its fellow. The ostia of both arches are guarded by semi-lunar valves, that of the left being situated to the right and below that of the right arch. As the *cavum arteriosum* is without an arterial trunk, red blood can be driven out only into the *cavum venosum* and during systole. The modus operandi of the heart is as follows :

With systole of the ventricle, the pulmonary artery and the aortic arches first receive venous blood from the *cavum venosum* and *cavum pulmonale* ; but as the red blood of the *cavum arteriosum* is driven into the *cavum venosum,* the venous blood of the latter is excluded from the mouth of the aortic arches and forced into the *cavum pulmonale,* when the arches receive only arterialized blood ; the left arch of course receives a larger proportion of venous blood than the right. Now, as the ventricle contracts, the free edge of the muscular septum approaches the dorsal wall and gradually bars all access to the *cavum pulmonale,* which thus finally expels the venous blood it has received from the *cavum venosum* without admitting arterialized blood, consequently the latter is not returned to the lungs. The vena cava, of course, pulsates simultaneously with the common ventricle and alternately with both auricles. Though but a three-chambered heart, by its own action it becomes in a sense a heart of *five* chambers.

Respiration is carried on with little activity, since creatures of such low organism consume but little oxygen, and may be deprived of it for considerable time without danger of asphyxia ; especially is this the case in cold weather, since temperature exerts no inconsiderable influence upon the respiratory function. With certain reptiles the skin absorbs oxygen, and in some few instances cutaneous transpiration is sufficiently active to prolong life indefinitely; but this is in no great degree true of *Testudinata.*

The lungs are each traversed by a single bronchus with lateral apertures opening into saculated pouches exhibiting a minimum of vascular surface for the absorption of oxygen, which accounts in no slight measure for the dilatory respiration. Neither are the lungs lodged in a thorax proper, since a true diaphragm is wanting; but, super-imposed to the viscera and cardiac apparatus, they extend nearly the whole length of the carapace and are fixed against its internal periosteum by reflected peritoneum. Of necessity, the parietes of the cavity being immovable, the mechanism whereby oxygen is supplied must differ from that in creatures possessed of a dilatable and distensible thorax, consequently air is brought in contact with the pulmono-vascular surfaces by action of the throat and mouth. The jaws being closed, the hyoid bone is lowered to enlarge the posterior oral cavity and upper pharynx, and air admitted through the nasal apertures. Next, the posterior nares are closed, the hyoidean apparatus is again hoisted into place as in the act of swallowing, and the air thus enclosed is now forced through the trachea to the bronchi. It is in fact a species of deglutition, though very far from the description given from the lecture platform by a well known naturalist, wherein it was asserted "air is swallowed and taken into the stomach, which serves as a reservoir, being subsequently forced to the lungs by muscular action as required."

The liver is merely a double "pancake" organ, the two lobes connected by a pair of narrow transverse bands; and the gall-bladder appears as a mere cyst enclosed by a proliferation of the peritoneo-hepatic membrane that binds it to the exterior or face of the right lobe, there being neither fissure or indentation for its reception.

A narrow flat pancreas is seen closely attached to the duodenum; in a large female it appeared fully four inches in length, and in a medium sized male was but a trifle shorter. In both the spleen appeared of equal size and shape, and as a cylindrical organ slightly flattened posteriorly, one and three-fourths inches in length, and varying in transverse diameter from four to six lines.

Numerous lobes overlapping each other compose the kidneys which, as with all *Vertebrata* with three chambered hearts, excrete nearly solid urine ; a peculiarity of turtles is a renal portal circulation receiving a major portion of the venous blood from the upper posterior portion of the body, including the posterior extremities and tail—the portal circulation of the liver receives blood only from the anterior abdominal veins, which are ıepresented in this class of reptiles by two trunks.

The ureters terminate in the common cloaca entering the bladder half or three-quarters of an inch below the vesical orifice, and in the female are adherent to the nipple-like processes of the oviduct, as in *Monotremata**.

The bladder, always muscular and of uniform oval outline, usually contains one or more calculi. Recently, to my surprise, in a well known medical periodical, I stumbled across an account of a calculus taken from the vesca of one of the *Emydidæ*, the circumstance being treated as astonishing and anomalous. Personal experiences with *Amyda* and *Aspidonectes* lead me to believe, however, that the absence of such concretions is more frequently the *exception* than the rule, and two opportunities to examine *Chelonia* served to confirm this opinion.

The generative organs of the male are disproportionately large, both in structure and form, which, however, is in

*Among the corrections and interlineations occurring in my notes in the handwriting of Doctor Sager I find the following query : "Does not such coincidence, including the cloaca, interior of oviducts, relations of uteri, and the growth of spines from the soft epidermis of this turtle, indicate positive affinity to *Echidna* ?"

At the time when this was penned the science of evolution was in its infancy, and the relations of the various orders of Vertebrata, one to another, little understood. Now, we see nothing wonderful in the semblance, since birds and reptiles are closely allied, and Monotremes are one of the first of the chain connecting oviparous and viviparous creatures. Recently it has been discovered that monotremes are *oviparous mammalia*, and thus a step lower in the Zoological scale than Marsupials. It is Echidna that has an affinity for reptilian forms, instead of the turtle an affinity to Echidna.

some measure accounted for by an examination of the cloaca in both sexes. In a specimen twelve inches in length, the penis is between four and five inches long in its relaxed condition, and eight or nine lines in diameter, its posterior portion provided with two soft diverging, erectile bulbs, the erectile tissue being continued the whole length of the corpora cavernosa and on into the *glans*, constituting the major portion of that extremely unique and complicated structure. T. Rymer Jones, remarking upon the intromittent organ as found in *Chelonia*, describes it as "very large" and "resembling that of the ostrich." He further adds: "It is long, nearly cylindrical in shape, and enlarged toward the extremity, which terminates in a point. A deep groove extends along the whole dorsal surface becoming gradually deeper as it approaches the glans, near the middle of which it terminates by a kind of orifice divided into two by papilla." The accounts of other authors are substantially the same.

But neither *Aspidonectes* or *Amyda* possess a pointed penis. Instead of the typical glans is found a double structure, each lateral moiety of which is sub-divided or bifurcated, and between a central isolated portion somewhat thicker than the surrounding sub divisions, and fully their equal in length. In other words it appears as a quinquefid structure. (In *Amyda* the central azygos is less prominent, and the glans partakes more nearly of a quadrifid nature.) Also the deep groove described by Jones upon the dorsal aspect of the organ, in this instance, separates into four on reaching the glans, corresponding to the four lateral divisions. Indeed the organ presents a striking resemblance to that of *Echidna aculeata* and of *Ornothorynchus anatinus*,* and some of the Marsupials.

---

*Dr. Sager has added the following: "It also presents striking affinity to the double bifurcated penis found in venomous *Ophidia—Viperinæ* and *Crotalidæ*—but is even more complicated and comprehensive than in these reptiles. Owen supposes the glans penis to be an adaptive structure, corresponding to the uteri of females of the same class, and with immediate reference to multiparous reproduction, but if such were true, we should find a quadruple uterus in *Trionychidæ* instead of the primitive double organ that exists. Is not the teleological significance rather suggested by the highly erectile structure of this part of the organ, and connected with the duration of the copulative act?"

The male organ is controlled by a pair of retractors, and a single protractor. The latter is a slender muscle arising from the sides of the fourth caudal vertebra, ascending into the pelvis, where it goes to be inserted in the dense fascia of the penal bulb. The former, instead of being attached to the pelvis as described by Rymer Jones and Ludwig Heinrich Bojanus, arise from the fourth dorsal vertebra, passing backward behind a broad flexor caudæ ischiadicus, in their course extending beneath the sheath of the penis, and beyond to be inserted into the central azygos of the glans. The peritoneal canals also, which in *Chelonia* are described as penetrating to the glans, here do not reach one-third the length of the intromittent member.

In a turtle, eight and one-half inches long, the testes were equally developed with those of another four inches larger, in both instances measuring four by eighteen lines, resembling the elongate appendages of pennibranchiate reptiles rather than the oval glands of *Testudinnata* generally. The vas deferens enters the cloaca at the same point as the ureters, and the oviducts in the female, forming small but distinct papillæ. The epididymes, like the vas deferens, are extremely long and convoluted, and from June to November were found turgid with white semi-fluid semen that, under the microscope, was found overflowing with spermatozoa.

The clitoris of the female is developed to about one fifth the size of the male organ and is its direct analogue in connection and mobileness. The oviducts are long, with distinct sphincters near the proximate orifice; and the interior or distal third of each is abruptly narrowed to form a duct that appears as the analogue of the Fallopian salpinx of higher Vertebrata, the remainder corresponding in relative position and value to the uteri of Monotremes and Marsupials. Unaccountably, the lining membrane of these organs is remarkably white and smooth, and with the exception of occasional isolated mucous glands, without either crypt or follicle, which raises the question how the eggs of the species are provided with suitable protection. That they are so provided, however, is evidenced by the eggs themselves, since during June and July, they were several times obtained from the reptile in all their perfection, in one instance a half dozen or more.

The ovaries, five or six inches in length, and sometimes longer, appear merely as the thickened edge of the membrane (mesovarium) that bind to the kidneys and other viscera, and they are but little more dense than those of *Ranidæ*, the ova being produced only in the narrow strip in the outer part of the membrane.

With an account of one more anomaly I will bring this already too prolix paper to its conclusion. The *retrahens capitis et colli* of Bojanus and Cuvier is wanting, being replaced by a simple *retrahens cervicis.* The former is described as arising by its longer part, "from the ribs of the fifth and sixth dorsal vertebræ ; passing between the lungs to the sides of the anterior neck there to be inserted by fasiculi in the transverse apophyses of the third, fourth and fifth cervical vertebræ ; a longer fasciculus passing on to the head to obtain insertion on the basilar bone ; the shorter part arising from the fourth and fifth dorsal beneath the articulation goes to be inserted in the side of the sixth cervical."

In *Aspidonectes* and *Amyda* the *retrahens cervices* takes its origin as a triceps. The long head arises from the posterior portion of the fourth caudal vertebra, passing up through the pelvis and along the spine ; the second is derived from the middle of the intercostal space of the sixth and seventh ribs ; the third from the outer edge of the seventh intercostal space ; converging, they retain distinct identity through the inter-pulmonary space, though lying side by side, coalescing in the anterior thorax to form a single tendon that is inserted at the side of the first cervical vertebra.

After many attempts at destroying the life of *Testudinata* for purposes of dissection, I have settled upon intravenous injection of chloral hydrate ; and this drug tends to preserve the soft heart, while arsenite of potassa, so justly celebrated for its effect upon morbid specimens in general, is not always to be relied upon. Catgut and wire ligatures applied to the neck and tightly twisted, produce no phenomena during thirty-six hours, save laborious action of the hyoidean apparatus; each attempt to force air to the lungs proving abortive, it escapes by the nostrils procuring a peculiar whistling note. During this time sensibility is little if any impaired, while pugnacity is apparently increased.

Forty-eight hours after severing the head the creature will yet wince under the application of the knife, and the motion of heart and bowels may be distinguished. Whether post-mortem sensitiveness is as marked as in *Chelydra*, I cannot say.

Dr. C. W. Larison remarks of *C. serpentaria*, examining seventy-two hours after decapitation, that upon attempting to draw up the left paw he "experienced a sensation that almost made my hair bristle. She contracted her fingers so forcibly, and at the same time flexed the forearm upon the arm with such force as to raise the entire remains from the table on which she lay." Ninety-six hours after decapitation the muscles of the legs still contracted when the derma was pricked or the toes touched. Twenty-four hours later the same occurred, and if the cauda was pricked the muscles contracted persistently; the sphincter cloaca contracted on the slightest provocation. One hundred and fifty-six hours after decapitation the cloacal orifice and the muscles of the tail still contracted when severely pinched, and during this time the creature had been deprived of its limbs, and eviscerated, leaving the cloaca alone intact. The heart continued to contract until removed (seventy-eight hours after decapitation and thirty-six hours after removal of the plastron) though it was "so dry that at each systole the exposed portion of the organ appeared quite wrinkled and husky."

